# Linkage to Care Is Important and Necessary When Identifying Infections in Migrants

**DOI:** 10.3390/ijerph15071550

**Published:** 2018-07-22

**Authors:** Manish Pareek, Teymur Noori, Sally Hargreaves, Maria van den Muijsenbergh

**Affiliations:** 1 Department of Infection, Immunity and Inflammation, University of Leicester, Leicester, LE1 7RH, UK; 2 Department of Infection and Tropical Medicine, University Hospitals Leicester NHS Trust, Leicester LE1 5WW, UK; 3 European Centre for Disease Prevention and Control, 16973 Solna, Sweden; Teymur.Noori@ecdc.europa.eu; 4 Section of Infectious Diseases and Immunity, Department of Medicine, Imperial College London, Hammersmith Hospital, London W12 0HS, UK; s.hargreaves@imperial.ac.uk; 5 The Institute for Infection and Immunity, St George’s, University of London, London WC1E 7HU, UK; 6 Department of Primary and Community Care, Radboud University Medical Center, 6525 GA Nijmegen, The Netherlands; Maria.vandenMuijsenbergh@radboudumc.nl; 7 Pharos, Dutch Centre of Expertise on Health Disparities, 3507 LH Utrecht, The Netherlands

**Keywords:** migration, health, infection, linkage, care

## Abstract

Migration is an important driver of population dynamics in Europe. Although migrants are generally healthy, subgroups of migrants are at increased risk of a range of infectious diseases. Early identification of infections is important as it prevents morbidity and mortality. However, identifying infections needs to be supported by appropriate systems to link individuals to specialist care where they can receive further diagnostic tests and clinical management. In this commentary we will discuss the importance of linkage to care and how to minimise attrition in clinical pathways.

Migration is an important driver of population dynamics in Europe with numbers of both internal (European Union) and external, both regular and irregular, migrants increasing over the last few decades [1]. Although the types of migrants arriving to Europe is highly diverse, in general, the majority of migrants are healthy [2]. However, there are subgroups of migrants arriving from low- and middle-income countries who bear a disproportionate burden of a range of infectious diseases—in particular HIV, tuberculosis (TB), multi-drug resistant TB, and hepatitis B and C (both undiagnosed and previously diagnosed but not treated). Whilst our understanding of infectious diseases and migrant health has improved, there is still a gap in the evidence-base relating to migrants in Europe. Nonetheless, the reasons for the higher prevalence of these infections in migrant populations appear to include, amongst many, a higher prevalence of infection in their countries of origin, low levels of screening and vaccination, increased levels of post-migration acquisition of certain infections (for example HIV) [3,4,5], barriers to healthcare on arrival, and low socioeconomic status [2]; however, further studies in European settings relating to the prevalence of infections and reasons for the higher prevalence (including sexual behaviour with respect to post-migration HIV acquisition) are required. As a matter of equity it is important that clinicians and policy-makers, in partnership with migrant communities, consider the specific and different needs of migrants when developing screening and vaccination programmes to reduce the burden of infection.

Identifying infectious diseases early is important as it mitigates adverse clinical outcomes and in some instances onward transmission; yet considerable heterogeneity exists across Europe as to how to approach migrant screening, and it is as yet unclear what represents a cost-effective approach [6,7]. Implementing migrant screening requires a clear understanding of how to screen migrants, where screening should happen (transit, arrival, or post-arrival) and the costs of having the testing done. In response, the European Centre for Disease Prevention and Control are developing evidence-based guidance on infectious disease screening for migrants [8]. This guidance, the product of intense work with key infectious disease experts across the European Union, will help to inform policy-makers and front-line clinicians on how to approach implementation of screening and vaccination in newly arrived migrants.

Integral to the development of the European Centre for Disease Prevention and Control (ECDC) guidance is an understanding of the importance of, and interventions for, each element of the care pathway from access to appropriate health services, to testing/screening, and then to follow-up treatment through to treatment completion and/or adherence.

We know from previous work relating to a range of infections that post-testing cascades of care occur with drop-out at each point in the clinical pathway including a failure to get results after testing, failure to attend specialist services to commence treatment, and failure to complete treatment [9]; data for migrants is less clear [10] but the principle remains the same—drop-outs in the post-screening/diagnostic testing services need to be minimised.

Drop-outs at each step of the care pathway (both in terms of attendance but also poor adherence to medication) can be caused by the plethora of personal and system-level barriers migrants may face in accessing statutory health/appropriate health services on arrival and subsequently, due to the lack of clarity about the organisation and financing of care compounded by linguistic and cultural barriers [11]. Many vulnerable migrant groups are not entitled to free statutory health care on arrival which will undoubtedly impact on uptake of screening and attendance at specialist services. Additional concerns for new migrants to European countries include competing psycho-social priorities such as housing, employment, concerns about family reunion, relationships, mental health issues, and chronic diseases. These problems not only interfere with testing, but also have the potential to increase the risks or consequences of infectious diseases. This synergistic interaction linked to socially disadvantaged circumstances, known as syndemics, calls for an integrated approach of public health and primary care, addressing biomedical as well as psychosocial problems [12].

Therefore, it is important that ease of access, making health services responsive, and engaging migrant communities is considered at an early stage when developing clinical pathways relating to screening for infection and appropriate vaccination. Engagement includes providing the necessary information and tailoring services to the needs and possibilities of the migrants involved [13]. Whilst this early work may seem less important, it likely sets in motion the basis for future community engagement, co-development of services, rapport and trust—particularly important when dealing with individuals who come from often marginalised and neglected communities.

Pathways of care should be designed with an understanding that the method in which the offer/rationale of testing is framed may well impact on whether individuals accept testing as well as how they view the subsequent result and, if necessary, follow-up with specialist care. Testing on its own achieves relatively little for the migrant with an infection if they do not attend follow-up and complete the necessary treatment. It is critical to always bear in mind that testing for infections is only one element of the migrants’ care pathway. A decision to test, by necessity, equates to an intention/decision to refer for assessment and, if required, treat. It is the linkage from testing to referral and attendance for specialist care which requires care and attention when designing care pathways and providing appropriate levels of education and information to migrants and front-line healthcare professionals. One specific area to consider when designing pathways of care is to consider how to make them as simple as possible for the migrant, with many professionals keen to move beyond working in infection silos (for example just screening for TB) but to consider multiple infections and vaccination, alongside other health needs. This will require working more closely with migrant communities to ascertain their view and concerns, but certain elements should be incorporated [14]:

1. Collaboration between primary care, public health, and specialist care in order to ensure continuity of care tailored to all the needs of the person involved.
2. Single point-of-referral to a migrant-friendly clinical service with culturally competent staff that deal with migrants and infectious diseases (as well as other health needs). This clinic could be facilitated by being staffed by specialists with a broad range of skill-sets who can manage all infections alongside interpreters and other support services to support treatment adherence and completion.
3. Robust data collection to facilitate sharing of best practice with respect to linkage to care and treatment completion for migrants with infectious diseases.

Although the patterns of migration across Europe are highly mixed, a lack of good quality data and limited sharing of best practice means that it remains unclear how best to deliver screening, vaccination, and treatment to migrants arriving in Europe. Evidence-based guidance emphasising methods of implementation supported by appropriate resources and migrant communities’ views have the potential to aid the design of stream-lined pathways of care for infectious diseases which address, and maximise, linkage to specialist care so that we have migrant services that meet the needs of a rapidly changing Europe.
